# Bayesian Nonparametric Modeling of Categorical Data for Information Fusion and Causal Inference †

**DOI:** 10.3390/e20060396

**Published:** 2018-05-23

**Authors:** Sihan Xiong, Yiwei Fu, Asok Ray

**Affiliations:** 1 Department of Mechanical Engineering, Pennsylvania State University, University Park, PA 16802-1412, USA; 2 Department of Mathematics, Pennsylvania State University, University Park, PA 16802-1412, USA

**Keywords:** Bayesian nonparametric, information fusion, causal inference, conditional tensor factorization, Bayes factor, sequential classification, thermoacoustic instability

## Abstract

This paper presents a nonparametric regression model of categorical time series in the setting of conditional tensor factorization and Bayes network. The underlying algorithms are developed to provide a flexible and parsimonious representation for fusion of correlated information from heterogeneous sources, which can be used to improve the performance of prediction tasks and infer the causal relationship between key variables. The proposed method is first illustrated by numerical simulation and then validated with two real-world datasets: (1) experimental data, collected from a swirl-stabilized lean-premixed laboratory-scale combustor, for detection of thermoacoustic instabilities and (2) publicly available economics data for causal inference-making.

## 1. Introduction

Modeling and decision-making in complex dynamical systems (e.g., distributed physical processes [1], macro-economy [2] and human brain [3]) often rely on time series collected from heterogeneous sources. Fusion of the information extracted from an ensemble of time series is a critical ingredient for better prediction and causal inference.

In many dynamical systems, the characteristic time of the physical process under consideration is small (e.g., around 2 ms in a typical combustion process) relative to the time-scale of respective decision-making (e.g., tenths of a second for active combustion control). Therefore, fast and accurate prediction of the system states and estimation of the associated parameters is essential for online monitoring and active control of the dynamical system; for example, real-time prediction of future states can significantly improve active control of thermoacoustic instabilities [4]. One way to achieve this is to make predictions based on different but correlated information sources. Although several methods have been proposed for prediction based on fusion of heterogeneous time series (e.g., [5,6,7]), they lack a coherent probabilistic interpretation and may not be able to accommodate more general interactions between current measurements and the measurement history. Furthermore, these methods may not be sequentially implementable and hence they may not be very useful for real-time applications.

Identification of causal relationships is essential for understanding the consequences of transitions from empirical findings to actions and thus forms a significant part of knowledge discovery. Various analytical techniques (e.g., [8,9,10]) have been proposed for causal inference-making; among these techniques, the concept of causality introduced by Granger [11], hereafter called Granger causality, is apparently one of the most widely used in time series analysis [12]. Granger causality does not rely on the specification of a scientific model and thus is particularly applicable to investigation of empirical cause-effect relationships. It is noted that Granger causality is especially suited for continuous-valued data based on frequentist hypothesis testing.

The goal of this paper is to develop a flexible and parsimonious model of categorical time series in a Bayesian nonparametric setting for fusion of correlated information from heterogeneous sources (e.g., sensors of possibly different modalities), which can be used for sequential classification and causal inference. From this perspective, major contributions of the paper are delineated as follows:

1. By introducing latent variables and sparsity inducing priors, a flexible and parsimonious model is developed for fusion of correlated information from heterogeneous sources (e.g., sensors of possibly different modalities), which can be used to improve the performance of sequential classification tasks.
2. By testing the dimension of latent variables in the setting of Bayes factor analysis [13], Granger causality [11] is extended to categorical time series.
3. Validation of the above concept with experimental data, generated from a swirl-stabilized lean-premixed laboratory-scale combustor [14], for real-time detection of thermoacoustic instabilities.
4. Testing of the underlying algorithm with public economics data to infer the causal relationship between two categorical time series.

The paper is organized into eight sections including the current section. Section 2 introduces the concept of Granger causality and develops the model. Section 3 discusses the algorithm for posterior computation using Gibbs sampling, and hypothesis testing using Bayes factor analysis. Section 4 presents the sequential classification algorithm with the proposed model. The underlying algorithms are tested with simulation data in Section 5 while Section 6 validates the proposed method with some experimental data, collected from a swirl-stabilized lean-premixed laboratory-scale combustor, for thermoacoustic instabilities early detection. Section 7 validates the proposed concept on publicly available economics data. Section 8 concludes the paper and provides a few recommendations for future research. The nomenclature and list of acronyms are provided at the end before the list of references.

## 2. Model Development

This section first introduces the concept of Granger causality and the corresponding regression model. Next, the underlying model’s algebraic and statistical specifications are elaborated.

**Definition** **1.** *(Granger Causality) Let ${\left\{y_{t}\right\}}_{t=1}^{T}$ and ${\left\{\theta_{t}\right\}}_{t=1}^{T}$ be two (statistically) stationary categorical time series. Then, the variable θ Granger-causes the variable y if the past values of θ contain statistically significant information for predictions of y besides those contained in the past values of y. Similarly, y Granger-causes θ if the past values of y contain statistically significant information for predictions of θ besides those contained in the past values of θ.*

**Remark** **1.** *The following are four types of Granger causality relationship between θ and y:*

*1*. *θ Granger-causes y but not the vice versa;*
*2*. *y Granger-causes θ but not the vice versa;*
*3*. *θ and y Granger-cause each other;*
*4*. *θ does not Granger-cause y and vice versa.*

However, in practice, only finitely many past values of *y* and θ are considered. To test the null hypothesis that θ does not Granger-cause *y*, the following regression model is constructed:

(1) $$p(y_{t}|y_{t-1},\cdots ,y_{t-D_{y}},\theta_{t-1},\cdots ,\theta_{t-D_{\theta }})\;$$

where in this model, predictors $y_{t-1}$ to $y_{t-D_{y}}$ represent variable *y*’s time lags; and predictors $\theta_{t-1}$ to $\theta_{t-D_{\theta }}$ represent variable θ’s time lags. In the sequel, for simplicity of notations, predictors $z_{t}\equiv \left(z_{1,t},\cdots ,z_{q,t}\right)$ are substituted for $\left(y_{t-1},\cdots ,y_{t-D_{y}},\theta_{t-1},\cdots ,\theta_{t-D_{\theta }}\right)$.

**Remark** **2.** *If the explanatory power of $\theta_{t-1},\cdots ,\theta_{t-D_{\theta }}$ to the regression is significant, then the null hypothesis (that θ does not Granger-cause y) is rejected and the alternative hypothesis (that θ Granger-causes y) is accepted. Hypothesis tests on the significance of time-lags are elaborated later in Equation (15) (see Section 3.2).*

**Remark** **3.** *If y and θ are correlated in the sense of Granger causality, the information contained in one source can be used to predict the future values in another source. Accordingly, information fusion of different sources enables fast and accurate prediction because of Granger-causality. It is noted that if the information contained in two sources is statistically independent, then information fusion cannot enhance prediction accuracy.*

### 2.1. Conditional Tensor Factorization

This subsection addresses fusion of different sources of information by making use of the concept of conditional probability tensor that was first reported in [15], a formal definition of conditional probability tensor follows.

**Definition** **2.** *(Conditional probability tensor) Let $C_{0}$ denote the number of categories of the (one-dimensional) variable, $y_{t}$, and let $C_{j}$ denote the number of categories of $z_{j,t}$ for j=1,⋯,q, where is the number of predictors. The quantity $p(y_{t}|z_{t})$ is treated as a (q+1)th order tensor in the $C_{0}\times C_{1}\cdots \times C_{q}$ dimensional space, hereafter called the conditional probability tensor.*

Let $C_{y}$ and $C_{\theta }$ respectively denote the numbers of categories of the variables, *y* and θ. It follows from Definition 2 that $C_{1}=\cdots =C_{D_{y}}=C_{y}$ and $C_{D+1}=\cdots =C_{q}=C_{\theta }$. Then, each one of these conditional probability tensors has a higher order singular value decomposition (HOSVD) of the following form [15]:

(2) $$p\left(y_{t}|z_{t}\right)=\sum_{s_{1}=1}^{k_{1}}\cdots \sum_{s_{q}=1}^{k_{q}}\lambda_{s_{1},\cdots ,s_{q}}\left(y_{t}\right)\prod_{j=1}^{q}\omega_{s_{j}}^{\left(j\right)}\left(z_{j,t}\right)$$

where $1\leq k_{j}\leq C_{j}$ for j=1,⋯,q; and each of the parameters $\lambda_{s_{1}\cdots s_{q}}\left(y_{t}\right)$ and $\omega_{s_{j}}^{\left(j\right)}\left(z_{j,t}\right)$ is non-negative while the following constraints are satisfied:

(3) $$\begin{array}{cc} \sum_{y_{t}=1}^{C_{0}}\lambda_{s_{1},\cdots ,s_{q}}\left(y_{t}\right) & =1,\;\mathrm{for}\;\mathrm{each}\;(s_{1},\cdots ,s_{q}) \end{array}$$

(4) $$\begin{array}{cc} \sum_{s_{j}=1}^{k_{j}}\omega_{s_{j}}^{\left(j\right)}\left(z_{j,t}\right) & =1,\;\mathrm{for}\;\mathrm{each}\;(j,z_{j,t}) \end{array}$$

**Remark** **4.** *Since there exists a factorization as in Equation (2) for each one of the conditional probability tensors, the two constraints Equations (3) and (4) are not restrictive. Furthermore, it is ensured that $\sum_{y_{t}=1}^{C_{0}}p\left(y_{t}|z_{t}\right)=\;1$.*

### 2.2. Bayesian Nonparametric Modeling

In order to build a statistically interpretable model, two techniques can be used to convert the tensor factorization in Equation (2) to a Bayes network, i.e., (1) introduce latent allocation-class variables; (2) assign sparsity-inducing priors. To this end, *T* pairs of variables and their respective predictors are collected in one dataset, and it is rearranged as ${\left\{y_{t},z_{t}\right\}}_{t=1}^{T}$, where *t* is an index with range from 1 to *T*.

The conditional probability $p(y_{t}|z_{t})$, factorized as in Equation (2), is then reorganized in the following form:

(5) $$p\left(y_{t}|z_{t}\right)=\int_{x_{1,t}}\cdots \int_{x_{q,t}}p\left(y_{t}|x_{t}\right)\prod_{j=1}^{q}p\left(x_{j,t}|z_{j,t}\right)$$

where $x_{t}\equiv \left(x_{1,t},\cdots ,x_{q,t}\right)$ denotes the latent class-allocation variables.

For index j=1,⋯,q and index t=1,⋯,T, it then follows that

(6) $$\begin{array}{cc} x_{j,t}|\omega^{\left(j\right)},z_{j,t} & ∼\mathrm{Mult}(\omega^{\left(j\right)}\left(z_{j,t}\right)) \end{array}$$

(7) $$\begin{array}{cc} y_{t}|\tilde{\lambda },x_{t} & ∼\mathrm{Mult}({\tilde{\lambda }}_{x_{t}}) \end{array}$$

where Mult(•) is the multinomial distribution [16] and $\omega^{\left(j\right)}\equiv {\left\{{\left\{\omega_{s}^{\left(j\right)}\left(c\right)\right\}}_{s=1}^{k_{j}}\right\}}_{c=1}^{C_{j}}$ is the mixture probability matrix. The *c*th row $\omega^{\left(j\right)}\left(c\right)\equiv {\left\{\omega_{s}^{\left(j\right)}\left(c\right)\right\}}_{s=1}^{k_{j}}$ in this mixture probability matrix is a probability vector itself (i.e., it sums to 1). Moreover, $\tilde{\lambda }\equiv {\left\{\lambda_{s_{1},\cdots ,s_{q}}\right\}}_{\left(s_{1}\cdots s_{q}\right)}$ is a conditional probability tensor where $\lambda_{s_{1},\cdots ,s_{q}}\equiv {\left\{\lambda_{s_{1},\cdots ,s_{q}}\left(c\right)\right\}}_{c=1}^{C_{0}}$ is a probability vector for each string $\left(s_{1},\cdots ,s_{q}\right)$.

The hierarchical reformulation of HOSVD above illustrates the following features of this model in Equation (5):

- Soft clustering for each one of the predictors $z_{j}\equiv {\left\{z_{j,t}\right\}}_{t=1}^{T}$ is implemented following Equation (6). This allows for inheritance of statistical strengths across different categories.
- The distribution of variable $y_{t}$ is determined by a probability tensor $\tilde{\lambda }$ of reduced order, following Equation (7).
- In order to capture the interactions among different predictors, class assignment variables $x_{j}\equiv {\left\{x_{j,t}\right\}}_{t=1}^{T}$ are used. They work in an implicit and parsimonious way by allowing the latent populations with the index of $\left(s_{1},\cdots ,s_{q}\right)$ to be shared across various state combinations of predictors.

**Remark** **5.** *Here it is very critical to distinguish these two different concepts: (1) the number of clusters ${\tilde{k}}_{j}$ generated by the latent class variables $x_{j}$ and (2) the dimensions $k_{j}$ of the probability vector $\omega^{\left(j\right)}\left(c\right)$ from the mixture probability matrix. The former one represents the number of groups generated by the data, and is smaller than the latter. It should be noted that ${\tilde{k}}_{j}$ determines if the predictor $z_{j}$ should be included in the model, because $p(y_{t}|z_{t})$ will not change with $z_{j,t}$ if $z_{j}$ has just a single latent cluster. Thus the significance of some particular predictor could be tested using on ${\tilde{k}}_{j}$, which is later elaborated in Section 3.2.*

In many real-world applications, the tensor $\tilde{\lambda }$ often has more components than needed, since the product $\prod_{j=1}^{q}k_{j}$ can be large even for modest values of *q* and $C_{j}$. To deal with this problem, tensor $\tilde{\lambda }$ is then clustered within different combinations of $\left(s_{1},\cdots ,s_{q}\right)$ nonparametrically by imposing a Pitman-Yor process prior [17]. Then, by using the stick-breaking representation of the Pitman-Yor process [18], it follows that

(8) $$\begin{array}{cc} \lambda_{l}|\gamma \; & ∼\mathrm{Dir}(\alpha ),\;\mathrm{for}l=1,\cdots ,\infty \end{array}$$

(9) $$\begin{array}{cc} V_{k}|a,b & ∼\mathrm{Beta}(1-b,a+kb),\;\mathrm{for}k=1,\cdots ,\infty \end{array}$$

(10) $$\begin{array}{cc} \pi_{l} & =V_{l}\prod_{k=1}^{l-1}\left(1-V_{k}\right),\;\mathrm{for}l=1,\cdots ,\infty \end{array}$$

where the bold symbols Dir(•) and Beta(•) represents the uniform Dirichlet distributions and Beta distributions [16] respectively, and $\lambda_{l}\equiv \left(\lambda_{l}\left(1\right),\cdots ,\lambda_{l}\left(C_{0}\right)\right)$. Moreover, 0≤b<1 and a>−b. For each combination $\left(s_{1},\cdots ,s_{q}\right)$, it follows that

(11) $$\phi_{s_{1},\cdots ,s_{q}}|\pi ∼\mathrm{Mult}\left(\pi \right)$$

where $\pi \equiv (\pi_{1},\pi_{2},\cdots )$. For t=1,⋯,T,

(12) $$y_{t}|\lambda \;,\phi ,x_{t}∼\mathrm{Mult}\left(\lambda_{\phi_{x_{t}}}\right)$$

where $\lambda \equiv {\left\{\lambda_{l}\right\}}_{l=1}^{\infty }$ and $\phi \equiv {\left\{\phi_{s_{1},\cdots ,s_{q}}\right\}}_{\left(s_{1},\cdots ,s_{q}\right)}$.

The next step assigns priors to the mixture probability matrix $\omega^{\left(j\right)}$. Here the dimension of $\omega^{\left(j\right)}$ grows linearly as $k_{j}$ increases (unlike the tensor $\tilde{\lambda }$). Therefore, further clustering of $\omega^{\left(j\right)}$ is not necessary. Hence, we assign independent priors to the rows of $\omega^{\left(j\right)}$ for j=1,⋯,q in the following way:

(13) $$\omega^{\left(j\right)}\left(c\right)|k_{j},\beta_{j}∼\mathrm{Dir}\left(\beta_{j}\right),\;\mathrm{for}c=1,\cdots ,C_{j}$$

Lastly, we assign priors to the dimension of the mixture probability vector $k_{j}$, i.e., for j=1,⋯,q,

(16) $$p\left(k_{j}=k|\mu_{j}\right)\propto \exp\left(-\mu_{j}k\right),\;\mathrm{for}k=1,\cdots ,C_{j}$$

where $\mu_{j}\geq 0$ and $k\equiv {\left\{k_{j}\right\}}_{j=1}^{q}$.

**Remark** **6.** *As the parameter $\mu_{j}$ grows larger, the exponential prior in Equation (14) will assign increasing probabilities to smaller values of $k_{j}$, and it becomes a uniform prior distribution on $\left\{1,\cdots ,C_{j}\right\}$ when $\mu_{j}$ is zero. Commonly, people have prior beliefs that as time lags increase, they will a have vanishing impact on the distribution of the current response variable. To impose this prior belief, we can assign larger $\mu_{j}$ to time lags further back in the history.*

**Figure 1:**
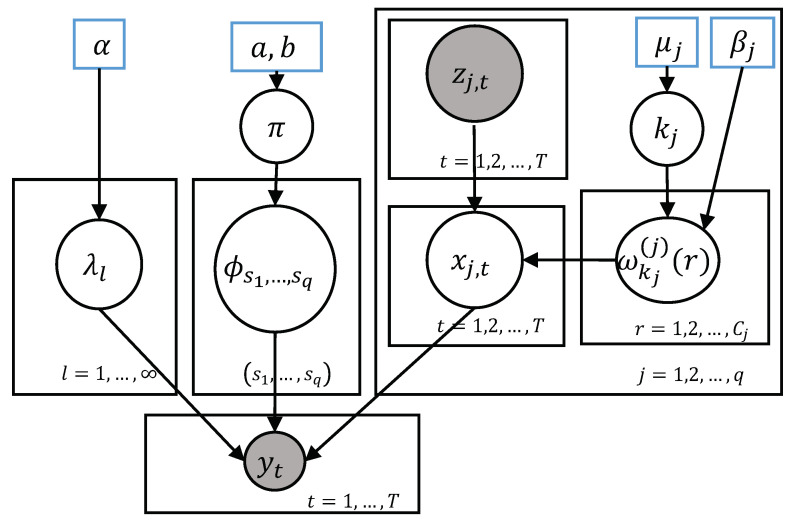
Bayes network representation of the model in the form of a graph. Deterministic hyperparameters are those that are enclosed by blue rectangles. Unobserved random variables are enclosed by transparent (unshaded) circles, and observed random variables are enclosed by shaded circles.

By combining Equations (6)–(14) together, a Bayes network representation of the model is created and Figure 1 illustrates its structures.

## 3. Estimation and Inference

  This section presents the details of an algorithm for computing posteriors as well as Bayesian hypothesis testing by using Bayes factors.

### 3.1. Posterior Computation

Despite the fact that the posterior distribution does not have any specific analytical form, we can still perform the inference of the corresponding Bayes network by using Gibbs sampling method. Because the dimension of $\omega^{\left(j\right)}$ may vary with $k_{j}$, constructing a stationary Markov chain by plain Gibbs sampling is difficult. To infer a model with variable dimensions, a common analytical tool—the reversible jump Monte Carlo Markov chain (MCMC) [19], which does trans-dimensional exploration in the model space—is often used.

Product partition modeling [20,21] can help alleviate difficulties occurring in trans-dimensional modeling by constructing a stationary Markov chain on the clustering space. For this proposed method, the dimension $\omega^{\left(j\right)}$ is being integrated out for the sampling of $k_{j}$ directly from $p(k_{j}|x_{j},z_{j})$, which will create a partially collapsed Gibbs sampler [22] that alternates between these two spaces: (1) the space with all the variables and (2) the space with all the variables but $\omega ={\left\{\omega^{\left(j\right)}\right\}}_{j=1}^{q}$.

To compute the posterior probabilities of the Pitman-Yor process, the infinite-dimensional tensors π and λ after their *L*th component are truncated, as performed in [18]. For achieving desired accuracy, an appropriate *L* needs to be chosen. Other than this, the posterior sampling is rather straightforward. The detailed process is presented in Algorithm 1, in which it is not explicitly mentioned that $x\equiv {\left\{x_{t}\right\}}_{t=1}^{T}$ and ξ collects the variables.

**Algorithm 1** Gibbs sampling for the proposed method
**Input:** Datasets ${\left\{y_{t},z_{t}\right\}}_{t=1}^{T}$; hyperparameters a, b, α, ${\left\{\mu_{j}\right\}}_{j=1}^{q}$, ${\left\{\beta_{j}\right\}}_{j=1}^{q}$; number of truncating components *L*; number of all samples *N*; initial sample ${}_{\left(0\right)}\phi ,{}_{\left(0\right)}\pi ,{}_{\left(0\right)}\lambda ,{}_{\left(0\right)}\omega ,{}_{\left(0\right)}x,{}_{\left(0\right)}k$.**Output:** All posterior samples ${\left\{{}_{\left(n\right)}\phi ,\;{}_{\left(n\right)}\pi ,\;\;{}_{\left(n\right)}\lambda ,\;{}_{\left(n\right)}\omega ,\;{}_{\left(n\right)}x,\;{}_{\left(n\right)}k\right\}}_{n=1}^{N}$
1:**for**n=1 to *N* **do**2:   For each one string $\left(s_{1},\cdots ,s_{q}\right)$, collect a sample $\phi_{s_{1},\cdots ,s_{q}}$ from its multinomial full conditional
$$p\left(\phi_{s_{1},\cdots ,s_{q}}=l|\xi \right)\propto \pi_{l}\prod_{c=1}^{C_{0}}{\left\{\lambda_{l}\left(c\right)\right\}}^{n_{s_{1},\cdots ,s_{q}}\left(c\right)}$$
   where $n_{s_{1},\cdots ,s_{q}}\left(c\right)=\sum_{1}^{T}1\left\{x_{1,t}=s_{1},\cdots ,x_{q,t}=s_{q},y_{t}=c\right\}$.3:   For l=1,⋯,L, update $\pi_{l}$ by the following rules
$$\begin{array}{c} V_{l}|\xi ∼\mathrm{Beta}\left(1-b+n_{l},a+lb+\sum_{k>l}n_{k}\right),\;l<L\\ V_{L}=1,\;\pi_{l}=V_{l}\prod_{k=1}^{l-1}\left(1-V_{k}\right) \end{array}$$
   where $n_{l}=\sum_{\left(s_{1},\cdots ,s_{q}\right)}1\left\{\phi_{s_{1},\cdots ,s_{q}}=l\right\}$.4:   For l=1,⋯,L, collect samples $\lambda_{l}$ from their respective Dirichlet full conditionals
$$\lambda_{l}|\xi ∼\mathrm{Dir}\left\{\alpha +n_{l}\left(1\right),\cdots ,\alpha +n_{l}\left(C_{0}\right)\right\}$$
   where $n_{l}\left(c\right)=\sum_{\left(s_{1},\cdots ,s_{q}\right)}1\left\{\phi_{s_{1},\cdots ,s_{q}}=l\right\}n_{s_{1},\cdots ,s_{q}}\left(c\right)$.5:   For j=1,⋯,q, for $c=1,\cdots ,C_{j}$, collect samples
$$\omega^{\left(j\right)}\left(c\right)|\xi ∼\mathrm{Dir}\left\{\beta_{j}+n_{j,c}\left(1\right),\cdots ,\beta_{j}+n_{j,c}\left(k_{j}\right)\right\}$$
   where $n_{j,c}\left(s_{j}\right)=\sum_{t=1}^{T}1\left\{x_{j,t}=s_{j},z_{j,t}=c\right\}$.6:   For j=1,⋯,q, for t=1,⋯,T, collect samples $x_{j,t}$ from their corresponding multinomial full conditionals
$$p\left(x_{j,t}=s|\xi ,x_{i,t}=s_{i},i\neq j\right)\propto \omega_{s}^{\left(j\right)}\left(z_{j,t}\right)\lambda_{\phi_{s_{1},\cdots ,s,\cdots ,s_{q}}}\left(y_{t}\right)$$7:   For j=1,⋯,q, collect samples $k_{j}$ from their respective multinomial full conditionals
$$p\left(k_{j}=k|\xi \right)\propto \exp\left(-\mu_{j}k\right)\prod_{c=1}^{C_{j}}n_{j,c}^{-k\beta_{j}},\;k_{j}=\max_{t}\left\{x_{j,t}\right\},\cdots ,C_{j}$$
   where $n_{j,c}=\sum_{t=1}^{T}1\left\{z_{j,t}=c\right\}$.8:
**end for**



To successfully run Algorithm 1, certain hyperparameters need to be chosen. The aforementioned determination of $\mu_{j}$ and *L* have been carefully discussed along with their implications, so we focus on the other hyperparameters. Among those hyperparameters, *a* and *b* will determine the clustering ability of the Pitman-Yor process (which are set to be 1 and 0 in this case), rendering it a Dirichlet process; this is sufficient for applications discussed in this paper. It should be noted that α and $\beta_{j}$ are Dirichlet Distribution’s hyperparamters and serve the role of pseudo-counts. The determination of these reflects the users’ prior belief. They are often manually chosen to be some small values without additional information which can justify larger values. In the following sections, they are chosen to be: α=1 and $\beta_{j}=1/C_{j}$ across different applications.

### 3.2. Bayesian Factor and Hypothesis Testing

This subsection discusses hypothesis testing techniques on the significance of all the predictors to the regression Equation (1). It can be used to make causal inference in order to provide a better understanding of the model and to better allocate computational resources for the sequential classification task by including only the important predictors (and discard the unimportant ones). As previously noted, a particular predictor $z_{j}$ is considered important if and only if the number of clusters ${\tilde{k}}_{j}$ formed by their corresponding latent class allocation variables $x_{j}$ is greater than 1.

Let Λ⊂{1,⋯,q} be the set of predictors under consideration. To perform the Bayesian hypothesis testing, we only need to compute the Bayes factor [23] in favor of $H_{1}:\;{\tilde{k}}_{j}>1$ for some j∈Λ against $H_{0}:\;{\tilde{k}}_{j}=1$ for any j∈Λ, given by

(15) $$BF_{10}=\frac{p\left(H_{1}|y,z\right)/p\left(H_{1}\right)}{p\left(H_{0}|y,z\right)/p\left(H_{0}\right)}$$

where $y\equiv {\left\{y_{t}\right\}}_{t=1}^{T}$, $z\equiv {\left\{z_{t}\right\}}_{t=1}^{T}$; and $p(H_{0}|y,z)$, $p(H_{1}|y,z)$ are numerically computed as the fraction of samples in which the ${\tilde{k}}_{j}$’s conform to $H_{0}$ and $H_{1}$, respectively; the prior probabilities $p(H_{0})$ and $p(H_{1})$ can be obtained by the following probability equation:

$$\begin{array}{cc} p({\tilde{k}}_{j}=1) & =\sum_{k=1}^{C_{j}}p\left(k_{j}=k\right)\sum_{l=1}^{k}p\left(x_{j,t}=l\;\forall \;t|k_{j}=k\right)\\ & =\left(\prod_{r=1}^{C_{j}}\gamma_{j}^{\left(n_{j,c}\right)}\right)\left(\sum_{k=1}^{C_{j}}\frac{p(k_{j}=k)k}{\prod_{k=1}^{C_{j}}{\left(k\gamma_{j}\right)}^{\left(n_{j,c}\right)}}\right) \end{array}$$

Specifically, to test whether θ Granger-causes *y*, it is only necessary to choose

$$\Lambda =\{D_{1}+1,\cdots ,q\}.$$

## 4. Sequential Classification

In Section 3, a Gibbs sampling algorithm is developed to infer the posterior distribution of model parameters given the observed data. In this section, a classification algorithm for dynamical systems based on the posterior predictive distribution, which is derived by marginalizing the likelihood of unobserved data over the posterior distribution of model parameters, is proposed. This algorithm consists of two phases: (1) off-line training phase and (2) online testing phase. Suppose there are *M* different classes of dynamical systems that are of interest, $C_{i},i=1,2,\cdots ,M$, for each of them we collect a training set ${}^{\left(i\right)}D{}_{T_{i}}={\left\{{}^{\left(i\right)}y{}_{t},{}^{\left(i\right)}z{}_{t}\right\}}_{t=1}^{T_{i}}$. The requirement for this dataset is that the data are categorical (e.g., quantized categories from continuous data), and for each class they have an identical number of categories of predictors and variables.

During the training phase, training set ${}^{\left(i\right)}D{}_{T_{i}}$ is used to compute the posterior of samples

$${\left\{{}_{\left(n\right)}^{\left(i\right)}\phi ,\;{}_{\left(n\right)}^{\left(i\right)}\lambda ,\;{}_{\left(n\right)}^{\left(i\right)}\omega \right\}}_{n=1}^{M}$$

for each one of the class $C_{i}$, as previously described in Algorithm 1. Then, during the test phase, the test set $D_{T}$ will be classified. Among these *M* classes, one will be identified as the class to which $D_{T}$ most likely belongs. In order to do so, the following conditional probability $p(D_{T}|{}^{\left(i\right)}D{}_{T_{i}})$ will be computed:

(16) $$\begin{array}{c} p\left(D_{T}|{}^{\left(i\right)}D{}_{T_{i}}\right)=\prod_{t=1}^{T}p\left(y_{t}|z_{t};{}^{\left(i\right)}D{}_{T_{i}}\right) \end{array}$$

(17) $$\begin{array}{c} \begin{array}{cc} & p\left(y_{t}|z_{t};{}^{\left(i\right)}D{}_{T_{i}}\right)\approx \frac{1}{N}\sum_{n=1}^{N}\left(\sum_{s_{1}=1}^{k_{1}}\cdots \sum_{s_{q}=1}^{k_{q}}{}_{\left(n\right)}^{\left(i\right)}\lambda {}_{{}_{\left(n\right)}^{\left(i\right)}\phi s_{1},\cdots ,s_{q}}\left(y_{t}\right)\prod_{j=1}^{q}{}_{\left(n\right)}^{\left(i\right)}\omega {}_{s_{j}}^{\left(j\right)}\left(z_{j,t}\right)\right) \end{array} \end{array}$$

Following the above calculation of conditional probabilities $p(D_{T}|{}^{\left(i\right)}D{}_{T_{i}})$, the posterior probability of the test data $D_{T}$ belonging to class $C_{i}$ (denoted as $p(C_{i}|D_{T})$) can be then calculated as:

(18) $$p\left(C_{i}|D_{T}\right)=\frac{p\left(D_{T}|{}^{\left(i\right)}D{}_{T_{i}}\right)p\left(C_{i}\right)}{\overset{M}{\sum_{r=1}}p\left(D_{T}|{}^{\left(r\right)}D{}_{T_{r}}\right)p\left(C_{r}\right)}$$

where $p(C_{i})$ is the prior probability of the class $C_{i}$. Next, the classification result is generated by:

(19) $$D_{\mathrm{class}}=\arg\;\max_{i}\;p\left(C_{i}|D_{T}\right)$$

The prior probability $p(C_{i})$ reflects user’s subjective beliefs and can also be designed to optimize some objective criterion. The reason that the detection algorithm is “sequential” is due to the fact the conditional probability $p(D_{T}|{}^{\left(i\right)}D{}_{T_{i}})$ is evaluated one by one as shown in Equation (16). In real-world applications, values of $p(y_{t}|z_{t};{}^{\left(i\right)}D{}_{T_{i}})$ in Equation (17) are often precomputed and stored for various values of $\left(y_{t},z_{t}\right)$, in order to achieve faster computations.

For the binary classification case, we can construct the likelihood ratio test [24] as:

(20) $$\frac{p(D_{T}|{}^{\left(1\right)}D{}_{T_{1}})}{p(D_{T}|{}^{\left(0\right)}D{}_{T_{0}})}\overset{1}{\underset{0}{≷}}\Theta$$

where in this equation Θ is a certain threshold. To choose the threshold Θ, one could rely on the receiver operating characteristic (ROC). ROC curves are often obtained by changing Θ in order to make a trade-off between the probability of (successful) detection $p_{D}=Prob\left(\mathrm{decide}\;\;1|1\;\;\mathrm{is}\;\mathrm{true}\right)$ and the false alarm probability $p_{F}=Prob\left(\mathrm{decide}\;\;1|0\;\;\mathrm{is}\;\mathrm{true}\right)$. Using those ROC curves, an optimal combination of $p_{D}$ and test set data length for a given $p_{F}$ can be selected, which would then determine the threshold Θ.

## 5. Numerical Example

This section presents a numerical example which utilizes the proposed method to infer causal relationships between two categorical time series. In this example, the data generation model is known and thus can be compared with the results from the proposed algorithm for evaluation of performance. The data generation details are given below.

In this particular numerical example, there are two binary sequences of symbols $y_{t}$ and $\theta_{t}$. Symbol sequences $y_{t}$ are generated using a known Markov model $p(y_{t}|y_{t-1},y_{t-3},y_{t-4})$, where only the time-lags $y_{t-1},y_{t-2},y_{t-5}$ are important predictors. Symbol sequences $\theta_{t}$ are generated from another Markov model $p(\theta_{t}|\theta_{t-1},\theta_{t-2},y_{t-1},y_{t-3})$, where $\theta_{t-1},\theta_{t-2}$ and $y_{t-1},y_{t-3}$ are the key predictors. In other words, the variable *y* Granger-causes the variable θ but not the other way around because *y* only depends on its own past. Table 1 lists the transition probabilities for $y_{t}$, where it is seen that the predictors are $y_{t-1},y_{t-3},y_{t-4}$ only. Table 2 lists the transition probabilities for $\theta_{t}$, where the predictors are $y_{t-1},y_{t-3},\theta_{t-1}$, and $\theta_{t-2}$ only.

To estimate the regression model in Equation (1) with the parameter T=1005, samples of ${\left\{y_{t}\right\}}_{t=1}^{1005}$ and ${\left\{\theta_{t}\right\}}_{t=1}^{1005}$ are being collected simultaneously. Based on the prior belief that $y_{t-D}$ and $\theta_{t-D}$ are no longer important for making predictions about $y_{t}$ and $\theta_{t}$ when *D* is greater than 5, predictors for both $y_{t}$ and $\theta_{t}$ are set as follows:

(21) $$z_{t}\equiv \left(y_{t-1},y_{t-2},y_{t-3},y_{t-4},y_{t-5},\theta_{t-1},\theta_{t-2},\theta_{t-3},\theta_{t-4},\theta_{t-5}\right)$$

From these data sets, 1000 training samples are chosen for testing the proposed algorithm.

**Figure 2:**
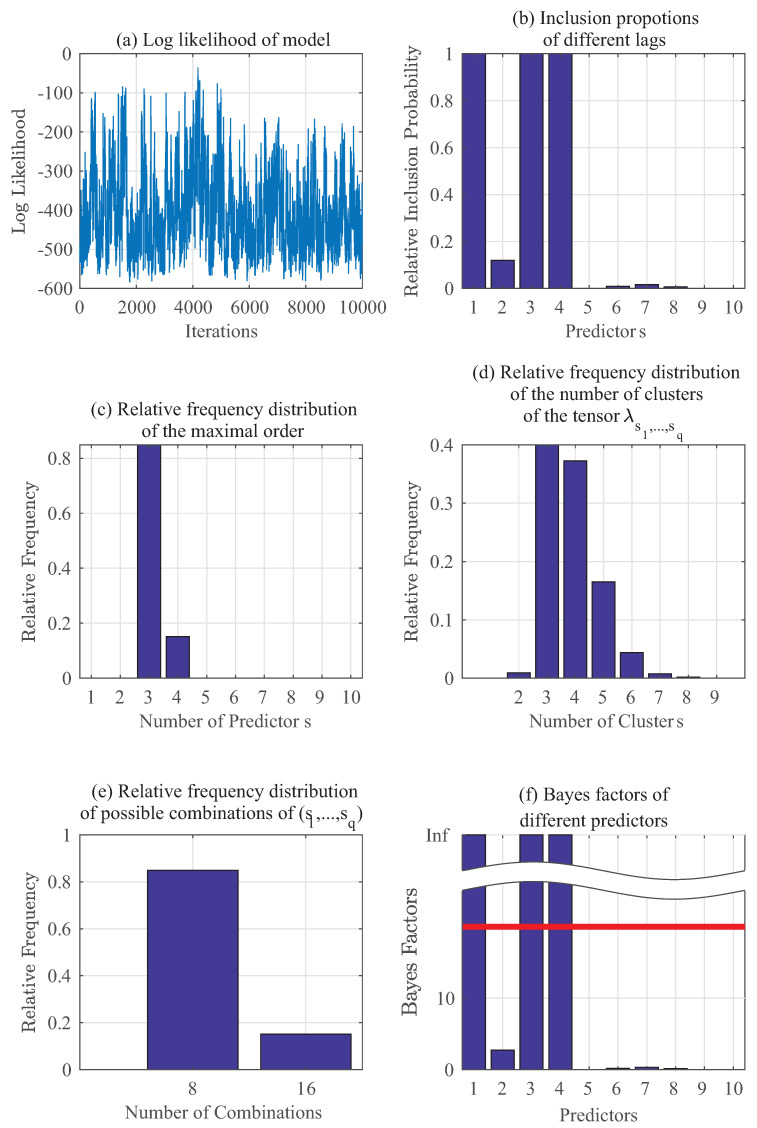
Gibbs sampling results: Numerical example for $p(y_{t}|y_{t-1},\cdots ,y_{t-5},\theta_{t-1},\cdots ,\theta_{t-5})$.

To calculate posteriors using Algorithm 1 for $p(y_{t}|z_{t})$, since there is no other prior knowledge, $\mu_{j}$ is set to be 1 across j=1,⋯,10. Initially, 200,000 samples are used in a burn-in period: they are fed into the algorithm and then discarded. The next 50,000 samples (after burn-in) are downsampled further by taking every 5th sample to reduce their autocorrelation. Figure 2 summarizes the results, in which Figure 2a displays the log-likelihood for 10,000 iterations of this model and Figure 2b illustrates the ability to correctly identify all the important predictors for the proposed method. For this example, the key predictors should be 1, 3 and 4, and the results from the prediction ($y_{t-1},y_{t-3}$ and $y_{t-4}$) are the same as the ground truth. Figure 2c shows the relative frequency of number of predictors that are important. Furthermore, the proposed method also creates parsimonious representations of the model as seen in Figure 2d,e. As previously discussed in Section 2.2, the tensor $\lambda_{s_{1}\cdots s_{q}}\left(y_{t}\right)$ has more components than needed but it can be clustered in a nonparametric way to reduce the number of combinations. Referring to [13], Figure 2f shows the Bayes factors calculation as mentioned in Section 3.2 for all of the predictors. Bayes factor $BF_{10}$ in Equation (15) can be regarded as the evidence against $H_{0}$. After setting a commonly-used threshold of t=20, it can be concluded that those predictors with higher $BF_{10}$ have implications of their evidences being strong. Furthermore, having $BF_{10}>150$ indicates even stronger evidence against the hypothesis $H_{0}$ [13]. It should be noted here that when the inclusion proportions of different lags in Figure 2b are equal to 1, then their corresponding Bayes factors in Figure 2f should tend to infinity (as for predictors 1,3 and 4 in this example).

**Figure 3:**
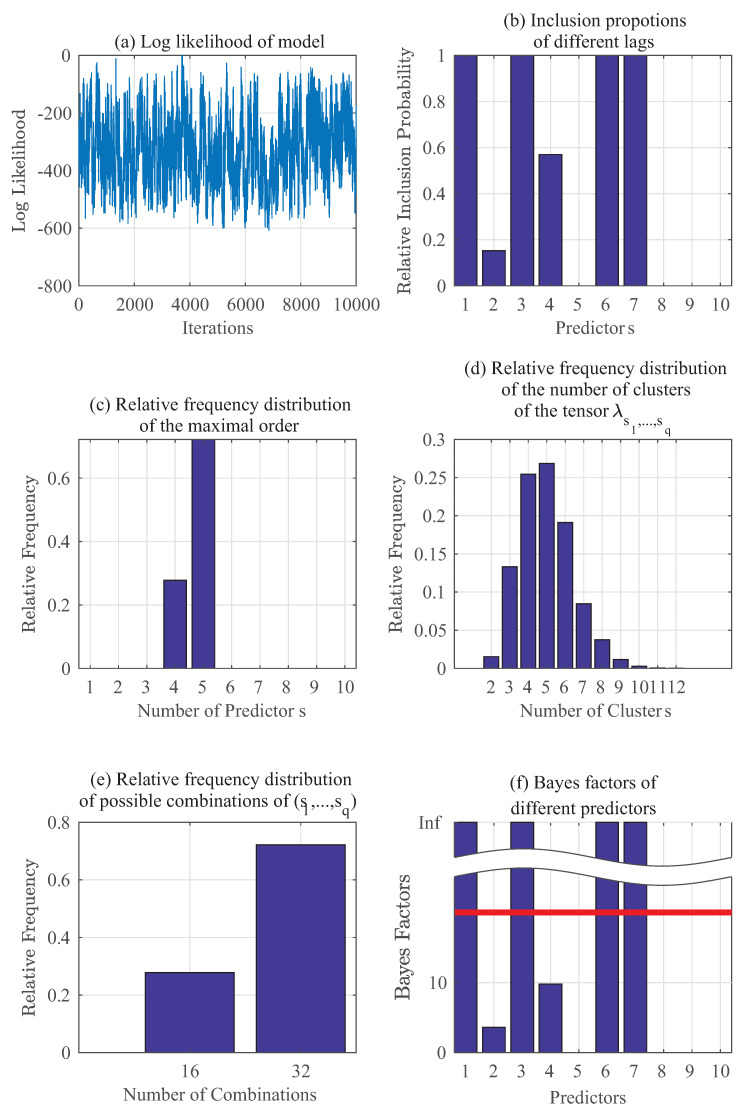
Gibbs sampling results: Numerical example for $p(\theta_{t}|y_{t-1},\cdots ,y_{t-5},\theta_{t-1},\cdots ,\theta_{t-5})$.

Similarly, Figure 3 shows the results using the same set of data as in Figure 2 but instead of estimating $p(y_{t}|z_{t})$, we are estimating $p(\theta_{t}|z_{t})$ here. Figure 3a–f have the same implications as those previously stated for Figure 2a–f. It can be seen that in this case for $p(\theta_{t})$, the key predictors should be 1,3,6 and 7, and the results confirm this in Figure 3.

**Figure 4:**
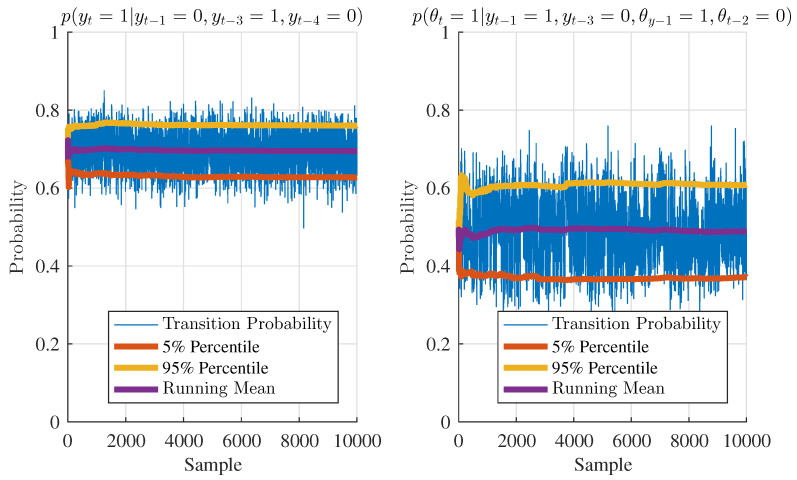
Transition probabilities in the numerical example.

Besides the ability to correctly identify the structure of the model, the proposed method can also perform transition probability estimation. Figure 4 illustrates two arbitrarily selected cases from Table 1 and Table 2. Setting $y_{t-1}=0$, $y_{t-3}=1$, and $y_{t-4}=0$, from Table 1 we can get the transition probability of the model of $\left(y_{t}=1\right)$ is 0.70. Similarly, setting $y_{t-1}=1$, $y_{t-3}=0$, $\theta_{t-1}=1$, and $\theta_{t-2}=0$, from Table 2 we can get the transition probability of $\left(y_{t}=1\right)$ is 0.50. In Figure 4, the estimated transition probability using the proposed method is displayed along with their running mean as well as their 5% and 95% percentiles. From both subplots of Figure 4, it is observed that the running mean of the transition probability is actually close to the true transition probability as given in the data generation tables. Even with a limited amount of data, the proposed method can not only estimate the transition probabilities, but also give an uncertainty bound in terms of their respective quantiles.

The causal relationship between y and θ is identified by Bayes factor analysis (see Section 3.2. The results are summarized in Table 3, which show that *y* Granger-causes θ but not the other way, which is in line with the ground truth.

## 6. Validation with Experimental Data: The Combustor Apparatus

This section validates the nonparametric regression model with experimental data generated from a swirl-stabilized lean-premixed laboratory-scale combustor apparatus [14].

### 6.1. Background and Description of the Experimental Procedure

This subsection presents a brief background of thermoacoustic instabilities in the combustor apparatus along with the experimental details for data collection. Thermoacoustic instabilities occur from highly nonlinear coupled phenomena that evolve from mutual interactions among thermofluid dynamics, unsteady heat release, and acoustics of the combustor chamber. The resulting self-sustained high-amplitude pressure oscillations often impose severe negative impacts on the performance and operational life of gas turbine engines [25,26,27].

Technical literature abounds with studies on combustion instabilities and their early detection by time series analysis, especially by using Markov chains [28,29]. However, current methods are largely limited to individual investigations of pressure or chemiluminescence measurements, and have apparently not taken the machine-learning-theoretic approach to information fusion into consideration; consequently, fast detection of thermoacoustic instabilities may not be achieved to the full extent based on the individual information of different sources only. Moreover, parameter estimation is difficult in current methods, even for moderately high-order Markov chains, due to the paucity of data, let alone a more sophisticated information fusion model. As for the detection procedure, empirical thresholds are often used in existing literature, without taking advantage of methods in statistical detection theory (such as sequential testing techniques); therefore, those applications are very limited in real-time detection cases.

**Figure 5:**
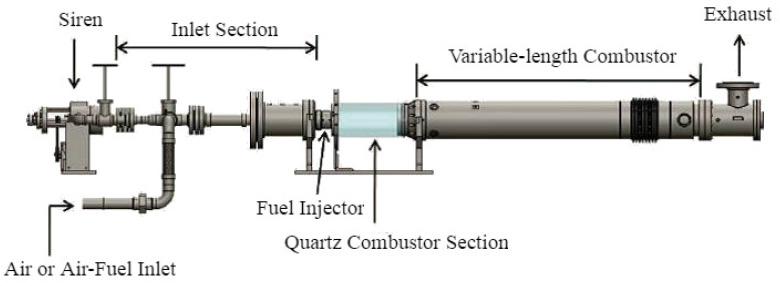
Schematic diagram of the combustor apparatus.

Figure 5 presents a schematic diagram of the combustor apparatus [14] that consists of an inlet section, an injector, a combustion chamber, and an exhaust section. The combustor chamber consists of an optically-accessible quartz section followed by a variable-length steel section.

Experiments have been conducted at 62 different operating conditions by varying the equivalence ratio and percentage of pilot fuel, as listed in Table 4. Under each operating condition, 8 s of pressure and chemiluminescence measurements have been collected at the sampling rate of 8192 Hz, where stable and/or unstable modes are recorded along with each time series data. To alleviate the problem of (possible) oversampling, the pressure and chemiluminescence measurements from combustors are first downsampled, which is obtained from first minimum of the average mutual information [30]. Then, the continuously varying time series data for both stable and unstable modes are quantized using maximum entropy partitioning [31,32] with a ternary alphabet Σ={1,2,3}. The quantized pressure measurements are denoted as $y_{t}$ and the chemiluminescence measurements are denoted as $\theta_{t}$ at time instant *t*.

### 6.2. Training Phase

This subsection describes details in the nonparametric regression model training, wherein 500 samples have been used after downsampling the quantized pressure time series data under stable and unstable conditions. The maximum memory *D* of each of $y_{t}$ and $\theta_{t}$ in this dataset is observed to be generally limited to 5 for both stable and unstable cases. Hence, predictors of $y_{t}$ or $\theta_{t}$ are set to be $z_{t}\equiv \left(y_{t-1},y_{t-2},\cdots ,y_{t-5},\theta_{t-1},\theta_{t-2},\cdots ,\theta_{t-5}\right)$ and the corresponding regression model is hereafter referred to as “full order model”. Since $y_{t}$ and $\theta_{t}$ has three categories, it follows that $C_{y}=C_{\theta }=3$.

To compute posteriors, as in Algorithm 1, the values

**Figure 6:**
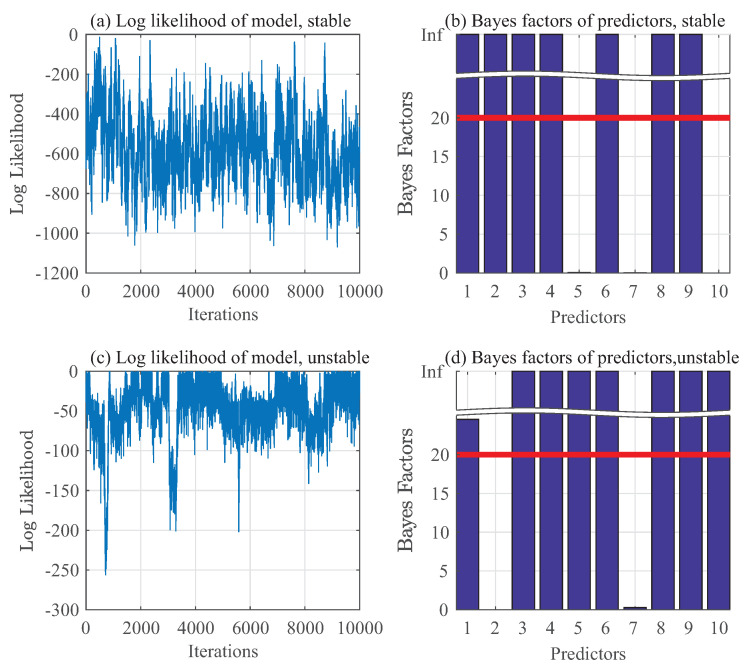
Gibbs sampling of pressure data.

**Figure 7:**
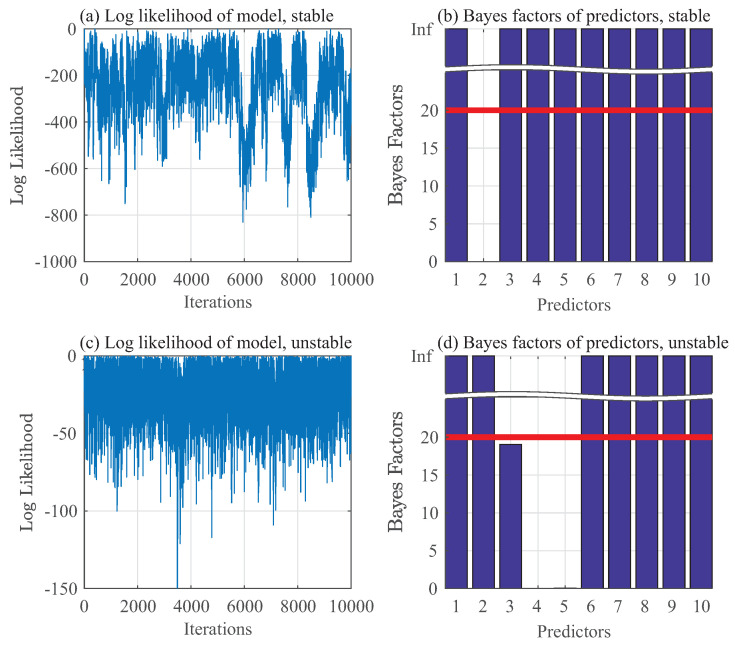
Gibbs sampling of chemiluminescence data.

[1,1.5,2.0,2.5,3.0,1.0,1.5,2.0,2.5,3.0]

are assigned to $\mu_{j}$ for j=1,⋯,10. After discarding 200,000 data points during the burn-in period, remaining 50,000 samples are then downsampled by taking every 5th data point to reduce their autocorrelation. Gibbs sampling results of pressure data are represented as $p(y_{t}|y_{t-1},\cdots ,y_{t-5},\;\theta_{t-1},\cdots ,\theta_{t-5})$ in Figure 6a,b for a stable mode and in Figure 6c,d for an unstable mode. Similarly, Gibbs sampling results of chemiluminescence data are represented as $p(\theta_{t}|y_{t-1},\cdots ,y_{t-5},\;\theta_{t-1},\cdots ,\theta_{t-5})$ in Figure 7a,b for a stable mode and in Figure 7c,d for an unstable mode.

Figure 6a,c and Figure 7a,c show the log likelihood with different iterations for pressure and chemiluminescence data under stable and unstable conditions, respectively. Similarly, Figure 6b,d and Figure 7b,d illustrate the Bayes factors of predictors for pressure and chemiluminescence data under stable and unstable conditions, respectively. Based on the Bayes factor analysis, the important predictors for stable pressure data are identified as:

$$y_{t-1},y_{t-2},y_{t-3},y_{t-4},\theta_{t-1},\theta_{t-3}\;\mathrm{and}\;\theta_{t-4}$$

while those for unstable pressure data are identified as

$$y_{t-1},y_{t-3},y_{t-4},y_{t-5},\theta_{t-1},\theta_{t-3},\theta_{t-4}\;\mathrm{and}\;\theta_{t-5}$$

**Figure 8:**
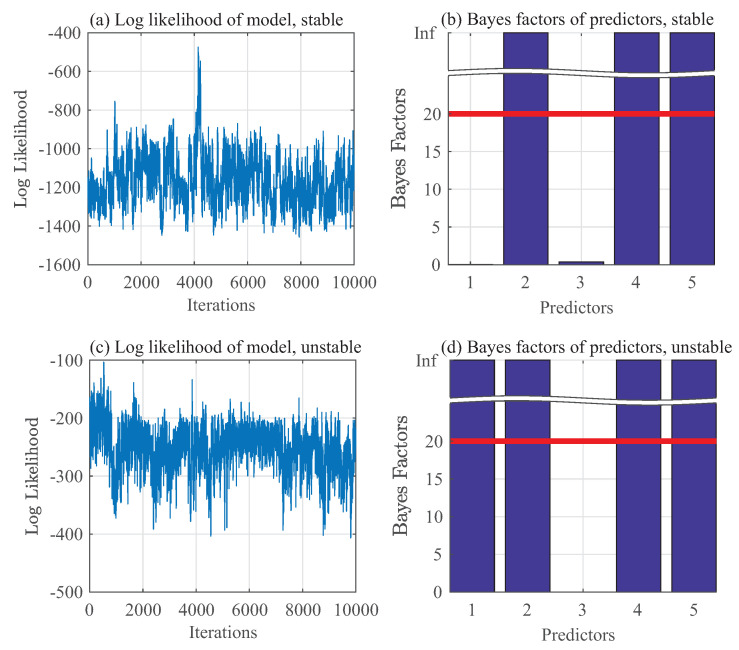
Gibbs sampling of the reduced-order model.

Using the identical set of hyperparameters and number of iterations, Gibbs sampling has been performed on the same set with pressure data $y_{t}$ only; this is referred to as the “reduced order model” in the following text. In this case the predictors are set as $z_{t}\equiv \left(y_{t-1},y_{t-2},\cdots ,y_{t-5}\right)$. The stable and unstable cases are shown in Figure 8a–d respectively. The important predictors for $y_{t}$ using this reduced order model are: $y_{t-2},y_{t-4}$, and $y_{t-5}$ for the stable mode, and $y_{t-1},y_{t-2},y_{t-4}$, and $y_{t-5}$ for the unstable mode.

Similarly, for chemiluminescence data, the important predictors are identified as:

$$y_{t-1},y_{t-3},y_{t-4},y_{t-5},\theta_{t-1},\theta_{t-2},\theta_{t-3},\theta_{t-4}\;\mathrm{and}\;\theta_{t-5}$$

while those for unstable chemiluminescence data are identified as:

$$y_{t-1},y_{t-2},\theta_{t-1},\theta_{t-2},\theta_{t-3},\theta_{t-4}\;\mathrm{and}\;\theta_{t-5}$$

### 6.3. Granger Causality

To identify the Granger causal relationship between pressure and chemiluminescence data, Bayes factor analysis has been performed for both stable and unstable cases as described in Section 3.2. The results are summarized in Table 5, which show that pressure and chemiluminescence measurements Granger-cause each other under both stable and unstable conditions; this implies that fusion of these two measurements can enhance the accuracy of prediction. This kind of mutual interaction between pressure and chemiluminescence measurements could be caused by a third unknown physical quantity, the exploration of which is a topic of future research.

### 6.4. Sequential Classification

**Figure 9:**
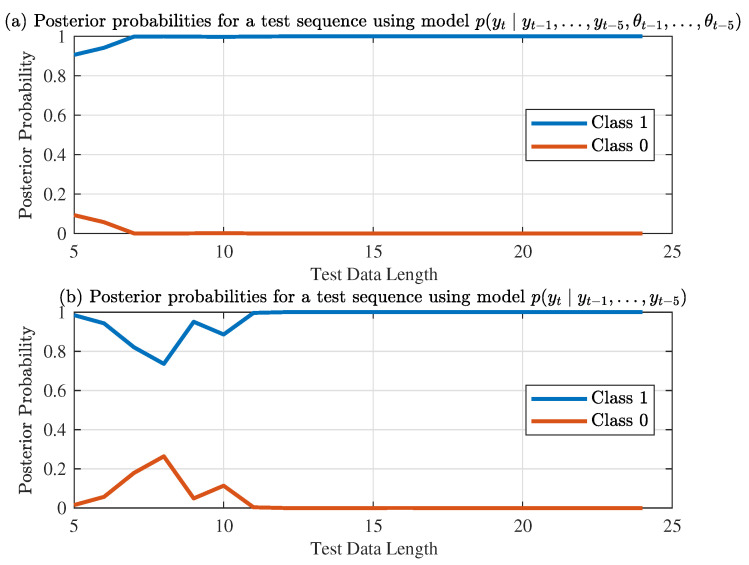
Posterior probabilities using different models.

For evaluation of the performance of the sequential classification for thermoacoustic instability identification, 100 instances of 50-sample datasets, which are not included in the training set, have been selected (also from their downsampled quantized pressure measurements for both stable and unstable modes). Figure 9 exhibits the profiles of posterior probability of each class as a function of the length of the observed data, where the top plate (i.e., Figure 9a) uses the full order model, and the bottom plate (i.e., Figure 9b) uses the reduced-order model for the same test data sequence. While the test sequences are correctly classified by both models, the reduced-order model is slower than the full-order model that contains more information.

**Figure 10:**
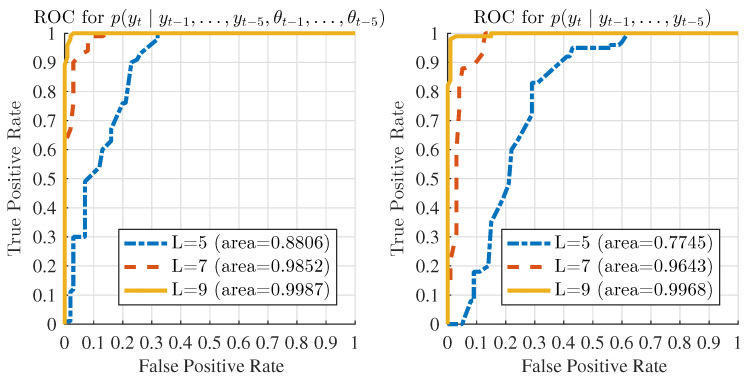
ROC curves with different test data length *L*.

Figure 10 shows the receiver operating characteristic (ROC) curves for the proposed detection algorithm with different lengths of the test data. These ROC curves are plotted for both full-order and reduced-order models to show that, when testing with the same dataset, the full order model achieves better detection performance in terms of the area under the ROC. In other words, the full-order model may achieve the same performance as the reduced order model in a shorter time, which is desirable for active control of thermoacoustic instabilities in real time. It is also observed that the ROC curves tend to improve (i.e., move toward the top left corner) considerably as the length of test data is increased from 5 to 9. This is expected because the information contents monotonically increase with the length of test data and hence better results are obtained.

## 7. Validation with Economics Data

This section validates the nonparametric regression model with (publicly available) real-world economics data. Specifically, monthly data of the U.S. consumer price index (CPI) and the U.S. Dollar London Interbank Offered Rate (LIBOR) interest rate index with one-month maturity from January 1986 to December 2016 are used. It is noted that: (i) U.S. CPI is a measure of the average change over time in the prices paid by urban consumers for U.S. market of consumer goods and services, and (ii) U.S. Dollar LIBOR is a benchmark for short-term interest rates around the world, which is not a monetary measure associated with any country, and which does not reflect any institutional mandate in contrast to, e.g., when the Federal Reserve sets interest rates. Economics theory [33] indicates that low interest rates can cause high inflation, and empirical research [34] has been conducted to investigate the causal relationship between inflation and nominal or real interest rates for the same country or region.

To avoid spurious regression [35], the raw data of U.S. CPI and U.S. Dollar LIBOR are preprocessed to achieve stationarity. U.S. CPI raw data are used to calculate the monthly percentage increase, and then this percentage increase is converted into a categorical variable by discretizing to quintiles (e.g., 5-quantiles in this study) that are denoted as $y_{t}$; the rationale for discretization of (noise-contaminated) continuously varying data is to improve the signal-to-noise ratio [36]. Similarly, U.S. LIBOR raw data are used to calculate the monthly difference, and then this difference is convertedin to a categorical variable by discretizing to quintiles, denoted as $\theta_{t}$. The entire dataset is used for training the proposed algorithm.

To estimate the regression model in Equation (1), based on the assertion that $y_{t-D_{y}}$ and $\theta_{t-D_{\theta }}$ are not important for predicting $y_{t}$ and $\theta_{t}$ if both $D_{y}$ and $D_{\theta }$ are greater than 6 (i.e, six months for both CPI and LIBOR), the predictor for $y_{t}$ and $\theta_{t}$ is set as:

(22) $$z_{t}\equiv \left(y_{t-1},y_{t-2},\cdots ,y_{t-6},\theta_{t-1},\theta_{t-2},\cdots ,\theta_{t-6}\right)$$

**Figure 11:**
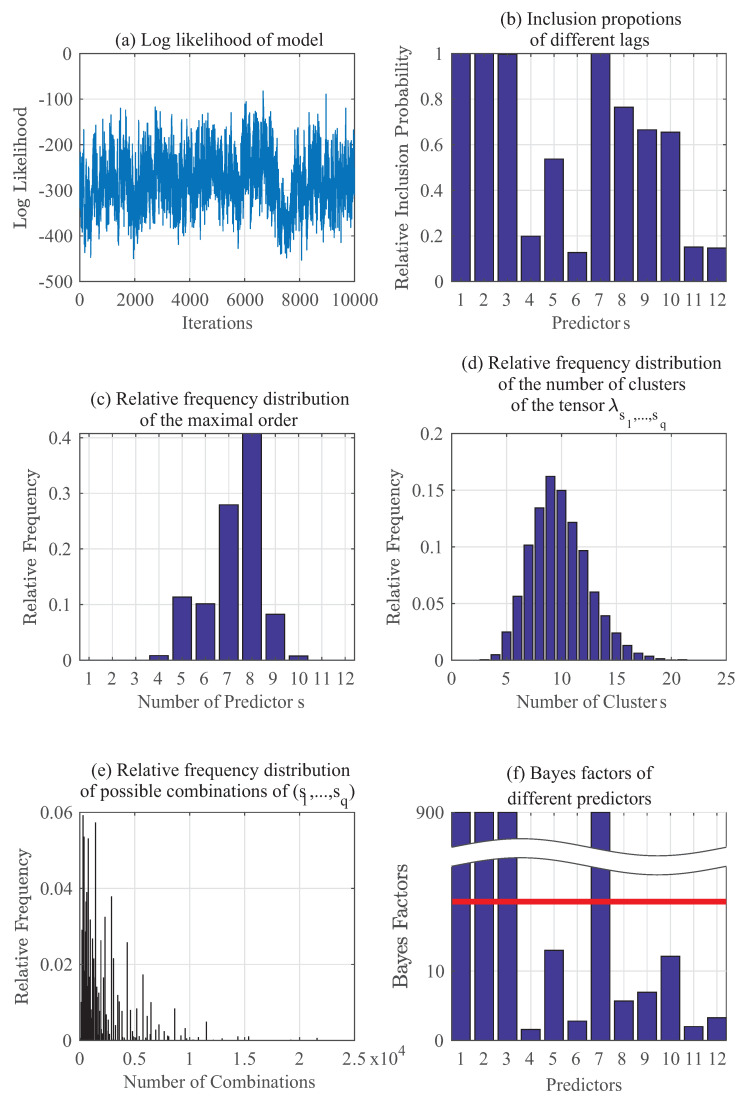
Gibbs sampling of economics dataset for $p(y_{t}|y_{t-1},\cdots ,y_{t-6},\theta_{t-1},\cdots ,\theta_{t-6})$.

**Figure 12:**
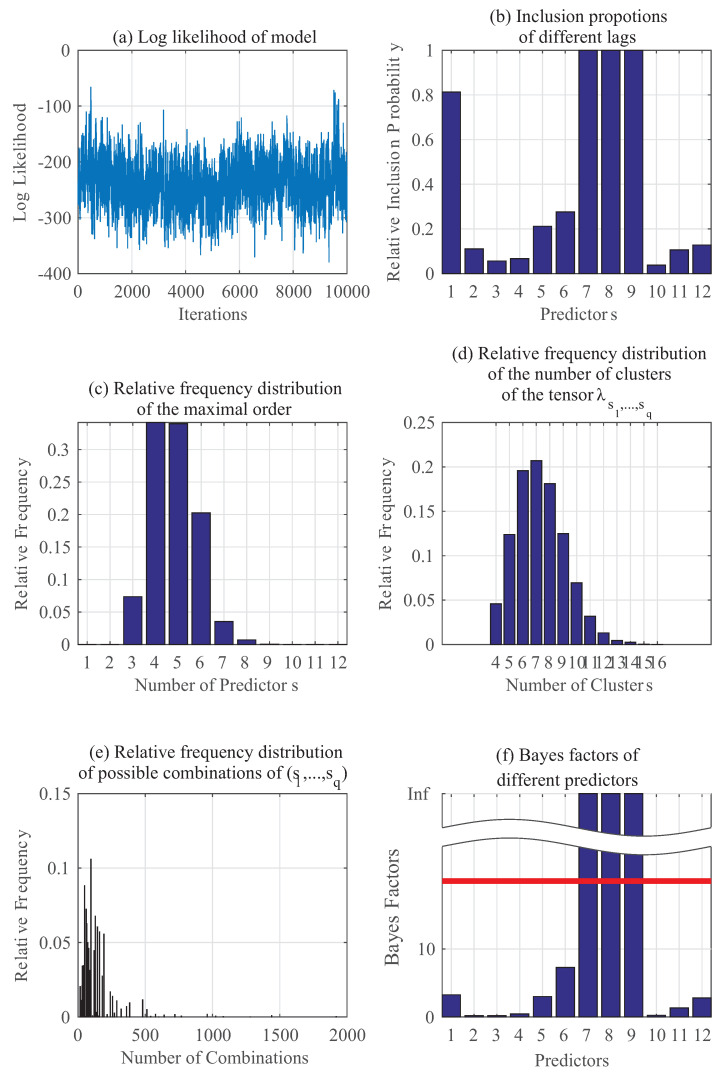
Gibbs sampling of economics dataset for $p(\theta_{t}|y_{t-1},\cdots ,y_{t-6},\theta_{t-1},\cdots ,\theta_{t-6})$.

To compute the posterior probabilities using the proposed Algorithm 1, $\mu_{j}$ are assigned to be j/2 for j=1,⋯,6 and (j−6)/6 for j=7,⋯,12. After the initial 100,000 samples are discarded during the burn-in period, the remaining 50,000 samples are then downsampled by taking every 5th to reduce their autocorrelation. Figure 11 and Figure 12 respectively summarize the results for $y_{t}$ and $\theta_{t}$. These figures have similar characteristics to their counterparts in the numerical example in Section 5. The results show that, for $y_{t}$ or CPI, the important lags are $y_{t-1},y_{t-2},y_{t-3}$ and $\theta_{t-1}$. Similarly, for $\theta_{t}$ or LIBOR, the important lags are $\theta_{t-1},\theta_{t-1},\theta_{t-3}$. These results show that LIBOR Granger-cause CPI, but not vice versa. This conclusion is summarized by Bayes factor analysis in Table 6.

## 8. Summary, Conclusions, and Future Work

The proposed Bayesian nonparametric method provides a flexible model for information fusion of heterogeneous, correlated time series data. The proposed method has been validated on a real-world application by using the experimental data collected from a laboratory-scale swirl-stabilized combustor apparatus, as well as on the publicly available economics data. It is demonstrated that the proposed method is capable of enhancing the accuracy for real-time detection of thermoacoustic instabilities and correctly identifying the Granger causal relationship between key economic variables.

There are many promising directions in which the proposed model can be further explored, such as:

1. Variational inference algorithm development for the proposed model [37].
2. Extension of the present analysis to hidden Markov models (HMM) [38] and information transfer [39].
3. Exploration of an unknown physical quantity that may cause the appearance of mutual interactions between pressure and chemiluminescence measurements.
4. Investigation of the empirical performance of the proposed approach utilizing extensive simulation studies.

## Figures and Tables

**Table 1:** Transition Probabilities for $y_{t}$ in the Numerical Example.

$y_{t-1}$	$y_{t-3}$	$y_{t-4}$	$p(y_{t}=1)$	$p(y_{t}=0)$
0	0	0	0.20	0.80
1	0	0	0.75	0.25
0	1	0	0.70	0.30
1	1	0	0.35	0.65
0	0	1	0.40	0.60
1	0	1	0.38	0.62
0	1	1	0.33	0.67
1	1	1	0.71	0.29

**Table 2:** Transition Probabilities for $\theta_{t}$ in the Numerical Example.

$y_{t-1}$	$y_{t-3}$	$\theta_{t-1}$	$\theta_{t-2}$	$p(\theta_{t}=1)$	$p(\theta_{t}=0)$
0	0	0	0	0.40	0.60
1	0	0	0	0.65	0.35
0	1	0	0	0.70	0.30
1	1	0	0	0.40	0.60
0	0	1	0	0.50	0.50
1	0	1	0	0.47	0.53
0	1	1	0	0.33	0.67
1	1	1	0	0.69	0.31
0	0	0	1	0.45	0.55
1	0	0	1	0.75	0.25
0	1	0	1	0.30	0.70
1	1	0	1	0.50	0.50
0	0	1	1	0.75	0.25
1	0	1	1	0.66	0.34
0	1	1	1	0.65	0.35
1	1	1	1	0.20	0.80

**Table 3:** Hypothesis Testing of Granger causality in the Numerical Example.

Null Hypothesis	Bayes Factor ${\mathrm{BF}}_{10}$
θ does not Granger-cause *y*	0.43
*y* does not Granger-cause θ	Infinity

**Table 4:** Operating conditions.

	Parameters	Values
**Variables**	Equivalence Ratio	0.525, 0.538, 0.575, 0.625
	Pilot Fuel (percent)	0–9% (0.5% increment)
**Fixed Conditions**	Inlet Temperature	$250{\;}^{\circ }C$
	Inlet Velocity	40 m/s
	Combustor Length	0.625 m

**Table 5:** Hypothesis Testing of Granger Causality.

Null Hypothesis	Operating Condition	${\mathrm{BF}}_{10}$
θ does not Granger-cause *y*	Stable	Infinity
*y* does not Granger-cause θ	Stable	Infinity
θ does not Granger-cause *y*	Unstable	Infinity
*y* does not Granger-cause θ	Unstable	Infinity

**Table 6:** Hypothesis test of Granger Causality for economics data.

Null Hypothesis	Bayes Factor ${\mathrm{BF}}_{10}$
US CPI does not Granger-cause LIBOR	7.29
LIBOR does not Granger-cause US CPI	Infinity

## Nomenclature of Pertinent Parameters

*a*	Hyperparameter of prior on probability vector π
*b*	Hyperparameter of prior on probability vector π
$C_{j}$	Number of categories of the *j*th predictor
$C_{i}$	*i*th class of dynamical systems
$D_{y}$	Number of time-lags of variable *y*
$D_{\theta }$	Number of time-lags of variable θ
${\tilde{k}}_{j}$	Number of clusters formed by $x_{j}$
$k_{j}$	Dimension of the *j*th mixture probability vector
k	Vector ${\left\{k_{j}\right\}}_{j=1}^{q}$
*L*	Number of truncations in a Pitman-Yor process
*N*	Number of iterations in Algorithm 1
*q*	Number of predictors
*s*	Realization of a latent allocation-class variable
*T*	Number of pairs of variables and predictors
$x_{j,t}$	*j*th latent allocation-class variables at time *t*
$x_{j}$	*j*th latent allocation-class variables ${\left\{x_{j,t}\right\}}_{t=1}^{T}$
$x_{t}$	Latent allocation-class variables ${\left\{x_{j,t}\right\}}_{j=1}^{q}$ at time *t*
x	Latent allocation-class variables ${\left\{x_{t}\right\}}_{t=1}^{T}$
$y_{t}$	Variable *y* at time *t*
y	Variables ${\left\{y_{t}\right\}}_{t=1}^{T}$
$z_{j,t}$	*j*th predictor at time *t*
$z_{j}$	*j*th predictors ${\left\{z_{j,t}\right\}}_{t=1}^{T}$
$z_{t}$	Predictors ${\left\{z_{j,t}\right\}}_{j=1}^{q}$ at time *t*
z	Predictors ${\left\{z_{t}\right\}}_{t=1}^{T}$
α	Hyperparameter of prior on λ
$\beta_{j}$	Hyperparameter of prior on $\omega_{j}$
$\theta_{t}$	Variable θ at time *t*
Θ	Threshold
$\lambda_{s_{1},\cdots ,s_{q}}$	Probability vector ${\left\{\lambda_{s_{1},\cdots ,s_{q}}\left(c\right)\right\}}_{c=1}^{C_{0}}$
Λ	Set of predictors
$\tilde{\lambda }$	Conditional probability tensor ${\left\{\lambda_{s_{1},\cdots ,s_{q}}\right\}}_{s_{1},\cdots ,s_{q}}$
$\lambda_{l}$	Probability vector ${\left\{\lambda_{l}\left(c\right)\right\}}_{c=1}^{C_{0}}$
λ	Sequence ${\left\{\lambda_{l}\right\}}_{l=1}^{\infty }$
$\mu_{j}$	Hyperparameter of prior on $k_{j}$
π	Probability vector ${\left\{\pi_{l}\right\}}_{l=1}^{\infty }$
ϕ	Collection ${\left\{\phi_{s_{1},\cdots ,s_{q}}\right\}}_{s_{1},\cdots ,s_{q}}$
$\psi^{\left(k\right)}$	Time-invariant spatial variables for *k*th experiment
$\omega^{\left(j\right)}\left(c\right)$	Mixture probability vector ${\left\{\omega_{s}^{\left(j\right)}\left(c\right)\right\}}_{s=1}^{k_{j}}$
$\omega^{\left(j\right)}$	Mixture probability matrix ${\left\{\omega_{s}^{\left(j\right)}\left(c\right)\right\}}_{c=1}^{C_{j}}$
ω	Mixture probability tensor ${\left\{\omega^{\left(j\right)}\right\}}_{j=1}^{q}$
**Pertinent Acronyms**	
BF	Bayes Factor
Beta	Beta Distribution
Dir	Uniform Dirichlet Distribution
HOSVD	Higher order singular value decomposition
Mult	Multinomial Distribution
ROC	Receiver operating characteristic
